# A Theory of Adaptive Intelligence and Its Relation to General Intelligence

**DOI:** 10.3390/jintelligence7040023

**Published:** 2019-10-01

**Authors:** Robert J. Sternberg

**Affiliations:** Department of Human Development, Cornell University, Ithaca, NY 14853, USA; robert.sternberg@cornell.edu

**Keywords:** intelligence, general intelligence, adaptive intelligence, analytical thinking, creative thinking, practical thinking, wisdom

## Abstract

Intelligence typically is defined as consisting of “adaptation to the environment” or in related terms. Yet, it is not clear that “general intelligence” or *g,* traditionally conceptualized in terms of a general factor in a psychometrically-based hierarchical model of intelligence, provides an optimal way of defining intelligence as adaptation to the environment. Such a definition of adaptive intelligence would need to be biologically based in terms of evolutionary theory, would need to take into account the cultural context of adaptation, and would need to take into account whether thought and behavior labeled as “adaptively intelligent” actually contributed to the perpetuation of the human and other species, or whether it was indifferent or actually destructive to this perpetuation. In this article, I consider the similarities and differences between “general intelligence” and “adaptive intelligence,” as well as the implications especially of the differences.

## 1. Introduction

The concept of intelligence has been defined or conceptualized in a number of different ways, some of them consistent with each other but others of them mutually contradictory [1,2,3,4]. There certainly is a real phenomenon to be explained, but people’s perceptions of that phenomenon differ quite radically, much as in the perceptions of a scene in the movies. For example, some see intelligence as a biologically defined trait [5], whereas others view it as a cultural invention whose biology would depend entirely on how a given culture chooses to define intelligence [6]. Some see intelligence as basically a unitary phenomenon [7], whereas others see it as a multiple phenomenon [8].

As Richard Haier has pointed out (see Note [5]), at some level, intelligence must be biological, in that even cultural variables that affected intelligence, such as various ways of bringing up children, would affect whatever biological substrate constitutes intelligence or how that substrate operates. But if researchers interested in intelligence are to understand intelligence biologically, they perhaps should begin not with the brain but rather with a different level of biology—the evolutionary function of intelligence, with different species showing different levels of biological adaptation [9,10].

## 2. Two Kinds of Intelligence

Consider two kinds of intelligence.

### 2.1. General Intelligence (General Cognitive Ability)

Almost all definitions of intelligence, for all their disagreements, agree on one thing—that intelligence crucially involves the ability to adapt to the environment (see [11] for a definition representing a consensus of experts). Sometimes, the language is different, as in [3] above: “*It reflects a broader and deeper capability for comprehending our surroundings—’catching on,’ ‘making sense’ of things, or ‘figuring out’ what to do*” (p. 13). But a common feature of the skills is that they involve adaptation to the environment. For example, skills such as “learning from the environment” or “reasoning about the environment” may be used in the wording of one definition or another, but one learns and reasons from and about the environment in order to adapt to it.

Without adaptation, one has little or nothing resembling intelligence. Imagine, for example, people who did well on intelligence tests created by scientists in a particular culture but who acted in ways that hastened their own demise and that of what might have been their future progeny. For example, perhaps they constantly started wars that regularly resulted in the genocide of their real or imagined opponents. Such actions would defy any rational and biologically-based Darwinian definition of intelligence as adaptation to the environment.

In the fictional tale of Superman, the people of Krypton ignored the warnings of a distinguished scientist, Jor-el, that their planet was about to explode. As a result, at least in the original version of the Superman tale, they all died (except for Jor-el’s son, Kal-el, who later became Superman on Earth). Whatever their scores on a Kryptonian intelligence test, allowing themselves to die en masse would seem contrary to any definition of intelligence as adaptation. Allowing a potentially avoidable mass extinction of one’s own species, as well as, of course, oneself, would seem like the ultimate act of biologically maladaptive behavior. Later I will argue that much current behavior on Earth is not so different from that on the fictional Krypton.

The principal argument of this article is that the term “intelligence” has come to be used to describe a construct that is related only vaguely, if at all, to intelligence as adaptation. Despite the definition of intelligence as adaptation, the usual use of the term has little to do with adaptation, at least in a biological sense. To be specific, the word “intelligence” has been used for more than a century to refer to a fairly standardized set of cognitive abilities, as first defined by Charles Spearman [12], elaborated upon by Louis Thurstone [13], and later further elaborated upon and systematized by John Carroll [14], among others. In this model, as Carroll posed it, and as is quite widely accepted today in an updated form distinct from what Carroll originally presented and called the CHC (Cattell–Horn–Carroll) model [15], intelligence is conceived of hierarchically, with *general intelligence* (g), or general cognitive ability (GCA), at the top of a hierarchy, with narrower abilities at each successive level of a hierarchy, conceived of in the Carroll and CHC models as having three levels (see also [16] for a discussion of the difference between the Carroll model and the CHC model). This is, more or less, the intelligence that intelligence tests measure [17,18]. It includes cognitive skills such as those involved in vocabulary acquisition and knowledge, inductive reasoning, spatial visualization, memory, and perceptual speed [19]. Different models involve different degrees of differentiation [20] or organization [21], but the skills highly overlap across models and across tests [22].

### 2.2. Adaptive Intelligence

In this article, I will discuss intelligence in what I believe is its true biological sense. My argument is not that intelligence is 100% biological—it is also affected by other variables at different levels of analysis, such as cultural ones. Rather, the argument is that whatever variables may affect intelligence, it is at its core a concept that concerns adaptive behavior. But in order to avoid confusion, I will refer to the proposed concept of intelligence as *adaptive intelligence*—intelligence that is used in order to serve the purpose of biological adaptation, which, for humans, always occurs in, and hence is mediated by, a cultural context. I will distinguish adaptive intelligence from the above-discussed general intelligence or general cognitive ability, which is the first (highest-order) factor in hierarchical models of cognitive abilities. I will argue that, in large measure, “general intelligence” in the sense we use it is largely culturally, not biologically, defined.

In biology, “adaptation” is a “process by which an animal or plant species becomes fitted to its environment; it is the result of natural selection’s acting upon heritable variation” [23]. Later, I will refer to this definition as referring to narrow adaptation.

There are other ways to look at biological adaptation. For example, a view of biological adaptation as egoistic might be to view it as preservation only of one’s own set of genes [24]. For the purposes of this article, it will not matter whether it is the species’ or one’s own gene pool that is put at risk. The arguments to be made will apply in either case.

General intelligence, as measured by Western psychometric tests and cognitive tasks, is not a necessary condition for adaptive intelligence across cultures. The reason is that, for many people, Western tests and tasks are not good measures even of general intelligence. This is not to say that no processes of intelligence are universal. I have argued that the processes of general intelligence, as illustrated by metacomponents, or executive processes, underlie all forms of intelligent behavior and are the same across cultures—recognizing the existence of a problem, defining the nature of the problem, mentally representing the problem, formulating a strategy to solve the problem, monitoring the strategy’s effectiveness during problem solution, and evaluating the effectiveness of the strategy after problem solution [25]. Moreover, certain kinds of information processing, for example, through working memory or sharing of attention, as described by current cognitive theories [26,27,28], certainly are highly relevant to understanding general intelligence across cultures. But how these processes play out “intelligently” can differ widely across cultural settings. IQ tests and cognitive tasks as we know them provide one way in which these processes can play out—as it happens, in the modern Western cultures of many of the readers of this article. However, as I have pointed out previously, and in some detail [29], although the processes are the same across cultures, the ways in which they manifest themselves and hence can be properly measured to be culturally relevant are not the same. The processes, as they are manifested in behavior, may differ greatly across cultures. On this view, it is important to distinguish between the processes of general intelligence and how those processes are measured, and to recognize that what we view as adequate measures in one place may not be adequate in another place. Different tests may be needed in different cultural settings accurately to measure general (or any other kind of) intelligence.

Our research has shown, for example, that practical and general (academic) intelligence are only very weakly correlated [30,31], even in the United States. If we extend this argument outward, to people of very different cultures, these people use these processes very differently from the way you or I do [32], and some of the high-IQ people of our society would and do look pretty stupid in the context of other cultures, such as ones placing a premium on hunting, gathering, ice-fishing, spatial-navigation, or other skills [33]. From this point of view, it is cultural hubris to suggest that the IQ-based skills as we measure and value them are somehow the fundamental ones. Place many of us even in an inner-city ghetto (or in an active war zone) overnight and we might not survive until the next morning to tell about our experiences, whereas ghetto children or children growing up in war zones, much younger than we are, likely would be there the next morning to tell the tale. Or as an example from our research among Yup’ik Eskimos, we found that even children in middle childhood could be placed in the winter in the frozen tundra and navigate a dog sled through scores of miles, without any obvious landmarks, from one Alaskan fishing village to another. Most of their teachers—and most of us—would die in the attempt [34].

The view of intelligence as biological adaptation in a narrow and a cultural sense oversimplifies intelligence. For example, cockroaches have apparently existed for roughly 300 million years, so they have been highly adaptive, at least at a species level. But if they are “intelligent,” it would seem to be in a more limited sense than the sense in which we usually refer to intelligence. In particular, I believe that a more capacious view of adaptation would be helpful in explaining why cockroaches, despite their narrow adaptivity, would not be viewed, biologically, as among the more intelligent species. In this regard, I have suggested that “adaptation” be broadly, as opposed to narrowly viewed as involving not only changing oneself to fit the environment, but also as changing the environment to fit oneself (shaping the environment) and finding or creating new environments as needed (selecting environments) [35]. Cockroaches are adaptive but their ability to shape and select environments, while not negligible, is far more limited than that of species with more adaptive intelligence. In the remainder of this article, I will be referring to adaptation not merely in the narrow sense of changing oneself to fit the environment but rather in the broad sense of additionally changing the environment to fit oneself and selecting new environments as necessary. Narrow adaptation, plus shaping and selecting of environments, is what I will refer to as broad adaptation.

The conundrum that arises from the definition of intelligence as adaptation (adaptive intelligence) is a result of two apparently incompatible outcomes: when (a) greater cognitive information-processing capacity (which is generally associated with higher general intelligence—see Notes [3,4]) is used (b) in ways that reduce adaptation (which is associated with lower adaptive intelligence). Consider some examples.

First, there is a consensus exceeding 95% among climate scientists that humans are acting in ways that exacerbate global climate change [36]. Carbon-dioxide emissions reached an all-time high in 2018 [37]. This human-induced climate change, in combination with other factors, is resulting in massive extinction of species, to the tune of one million species [38].

One could argue that intelligence as adaptation applies only to one’s own species. But the effects of climate change already are resulting in human deaths, for example, through rising heat, hurricanes, tornadoes, typhoons, and flooding resulting as well from other extraordinary weather events. It has been estimated that, in future decades, more than a quarter of a million people may die annually because of global climate change [39].

One might argue that general intelligence and related constructs are the only ones necessary to explain inaction on climate change. On this view, smart, scientifically-minded people believe in climate change and not so smart, scientifically illiterate people don’t. This view is incorrect, however. In fact, general intelligence and its correlates such as scientific literacy are poor predictors of views on climate change and related phenomena [40].

Were human-induced climate change a lone example of species-destructive behavior, one might view it merely as a one-off. Unfortunately, of course, it is a consequence of only one of many species-destructive behaviors exhibited by humans, many of whom are high in general intelligence.

Second, the creation of technology that produces enormous amounts of air pollution (which in turn is linked to climate change) is species-destructive. Air pollution has been linked to approximately 4.2 million human deaths annually, as well as to severe respiratory and other related illnesses [41]. To put the figure of 4.2 million into proportion, this number exceeds the population of the second largest city in the United States, namely, Los Angeles, California. As it happens, Los Angeles ranks as number one in ozone pollution among all the cities in the United States [42]. Were air pollution merely annoying, the situation would be unpleasant but not dismal. However, air pollution kills people and also results in stunting of cognitive growth of children [43]. Air pollution in India caused well over a million deaths in 2017 [44].

One could argue, of course, that problems with global climate change and air pollution have nothing to do with intelligence, but rather a lot to do with avarice, desire to promote one’s own interests at the expense of the interests of others, ideology, or short-term thinking. To the extent that one views intelligence in terms of general intelligence, this argument might indeed be largely correct. But if one is dealing with adaptive intelligence, the argument does not hold up, because adaptive intelligence by definition requires behaviors that are biologically adaptive in leading to species or genetic survival; creating an uninhabitable planet for ourselves, our children, and future generations beyond is definitely biologically maladaptive, no matter where such behavior falls on the general-intelligence scale, if it falls on that scale at all.

Third, one could go on with other species-extinguishing inventions, such as nuclear weapons, which killed over 100,000 people in Hiroshima and not much short of 100,000 in Nagasaki [45]. One could argue that killing those people was necessary to save more other people, but how far will this argument extend for potential future use of nuclear weapons by terrorists or rogue states, with the potential for many more to be killed in the future either by the explosions resulting from the weapons or by the “nuclear winter” that follows? 

Another, fourth impressive feat is that the same species, *Homo sapiens,* which has had members who were generally intelligent enough to create antibiotics also has members who are adaptively unintelligent enough to overuse them and render them ineffective, posing one of the greatest extant threats to global health [46]. Finally, there are anti-vaxers, many of them highly generally intelligent, whose opposition to vaccination of children, based on pseudo-science, has led to a recurrence of measles, a disease previously thought to be eradicated.

Humans appear to be acting, en masse, in ways that threaten their own adaptation. Some of the more generally intelligent ones are creating the technology that is destructive to the species, whether in the short run or the long run. How can one square the result of their behavior with the notion of intelligence as biological adaptation? It might seem paradoxical that greater general intelligence, and even 30 points of increase in IQ during the 20th century [47], would lead to behavior that ultimately is species self-destructive and thus lower in adaptive intelligence. How does one resolve the paradox? Several resolutions could be imagined.

## 3. Resolutions of the Paradox of Apparent High General Intelligence and Species-Maladaptive Destructiveness

High general intelligence and species-maladaptive destructiveness—low adaptive intelligence—could be viewed as compatible if one of several resolutions of the apparent paradox were proposed.

### 3.1. There Is No Paradox: General Intelligence Has Nothing to Do with Biological Adaptation

One argument would be that general intelligence simply is orthogonal to biological adaptation—that the one has nothing to do with the other. If one is all right with a species being so intelligent that it extinguishes itself, then perhaps this argument can hold up. The problem is that, sooner or later, there will be no one left to decide whether the argument was indeed valid. If the purpose of intelligent thinking—learning, remembering, reasoning, problem solving, decision making—is not adaptation to the environment, then what is its purpose? What is intelligence for, if not adaptation?

### 3.2. There Is No Paradox: General Intelligence Is about Individual, Not Collective Adaptation

One possibility is the assertion that general intelligence pertains not to adaptation at the species or other such level but rather to adaptation at the individual level. Thus, the argument would be that general intelligence is what enables an individual to adapt to his or her individual environment or set of environments. This seems to be the solution that has been taken by default by the field of general-intelligence research, where criteria tend to be related to individual performance, such as test scores, grades in school, individual performance ratings at work, and the like. However, this solution is problematical for several reasons.

First, as has become abundantly apparent through the literature on general intelligence, there is no clear definition of “individual adaptation”. The result has been a series of less than fully satisfactory criteria supposed to measured individual adaptation or success that have been used to validate general-intelligence tests. Criteria seem to have been chosen for ease of measurement, for convenience, and for utilitarian conformity to a Western sociocultural norm of success, as in this statement [48]: “*The measurement of intelligence—which has been done primarily by IQ tests—has utilitarian value because it is a reasonably good predictor of grades at school, performance at work, and many other aspects of success in life (Gottfredson, 2004; Herrnstein &Murray, 1994)*” (p. 131) (see also Notes [3,49]).

These and related criteria may be valued by people in our culture but may be less valued by people in other cultures, where adaptive tasks are different. Correlations of quality of behavior with general intelligence that are positive in our culture may even be negative in other cultures [50,51]. Thus, to state that the tests measure intelligence because they correlate with specific individual outcomes may tell us more about what kinds of behavior our culture values than about what intelligence is. In other cultures, skills in hunting, figuring out how to stay warm, fishing, gathering, avoiding parasites and larger predators, or building a safe dwelling might be far more important to intelligence as a matter of culturally-bound success. Indeed, skill in avoiding predators can become important unexpectedly, as when a hostile force takes over one’s place of dwelling (which has happened in many nations or nation-equivalents around the world, dating back to prehistoric times). The skills for avoiding the predators may be more linked to practical intelligence—tacit procedural knowledge for getting along in the world—than to the kind of analytical intelligence measured by typical intelligence tests [52,53]. The general point, though, is that external validation of intelligence tests draws upon culturally-valued criteria, and these criteria differ across cultures, as do the acts considered to be adaptively intelligent. Interestingly, general intelligence has increased over the years, whereas adaptive intelligence well may not have.

Flynn [54] related the increase of IQs (which are largely although not entirely proxy measures of general intelligence) during the 20th century to the increased abilities of people over the course of that century to adapt to the demands of a world that increasingly requires hypothetical questions about the world as it might exist. But not all cultures have viewed such hypothetical reasoning as important or even as adaptive. Further, culture matters a lot in terms of what makes a person adaptively intelligent. Luria, for example, demonstrated that Asian peasants in the former Soviet Union did not perform well on typical cognitive tasks requiring hypothetical reasoning because of the peasants’ refusal to accept the tasks as they were presented [55,56]. They did not want to engage in what Narvaez [57] called “detached imagination,” or reasoning with hypotheticals. Asked to imagine a state of the world different from the state of the world in which they live or conceivably might live, they simply rejected the reasoning task as silly and misguided. Some might view these people as not very intelligent. But those who do not think in such hypothetical terms may, in turn, have other skills that most of us today do not possess. Gladwin [58], who studied the Puluwat people inhabiting the Caroline Islands in the South Pacific, discovered that the individuals were totally able to master domains of knowledge relevant to their world, including weather and wind, ocean currents, and positions of the stars. They were able to integrate this knowledge with their mental maps of the islands to become skilled navigators who were highly competent and appropriately respected in the world in which they lived. In a related vein, what you and I might think of as sophisticated logical thinking—for example, sorting taxonomically (as in classifying a sparrow as being a kind of bird)—might be viewed as unsophisticated by the Kpelle [59,60,61,62]. Their functional performance on tasks requiring sorting into categories corresponded to the demands that were omnipresent in their daily life (as in a sparrow flying through the sky). Moreover, those of us living in technologically advanced societies have lost many of the skills that are present and even required by life in First Nation communities in Canada [63]. We fail to recognize natural patterns—of plants, wildlife, weather—that are apparent to them through what Gardner [64] would call their naturalist intelligence.

The examples above illustrate the importance not only of individual- and species-level adaptation, but also the importance of what might be viewed as a tribal level of adaptation. One might have thought that World War II would have shown the potential horrors of tribalism, but today, tribalism appears to be back with a vengeance, for example, in politics [65] and even in academia [66]. Today, tribalism is still encouraged, especially by toxic political leaders seeking to enhance their power by pitting groups against each other and even by encouraging one (often imagined) tribe against another [67]. Psychometric tests do not capture the non-adaptive elements of the tribalism to which even generally intelligent academics are susceptible (e.g., in their pecking orders for different academic disciplines or subdisciplines).

One might be tempted to view a typical psychometrically-based approach to validating intelligence tests as somehow more “objective” and detached than an approach that takes culture into consideration. But the criteria used to psychometrically validate intelligence have their own issues, not only of objectivity, but of whether they truly show validity. Consider some of these criteria that are commonly used in test validation.

One criterion commonly used to validate general-intelligence tests—correlating scores on a new general-intelligence test with scores on old general-intelligence tests—is problematical. Such a procedure would assume the operational definition that it is trying to validate, namely, that intelligence tests—any of them—actually measure intelligence. The circularity of Edwin Boring’s definition of intelligence as what intelligence tests measure [68] has been pointed out many times [69,70]. In this case, one creates tests to measure intelligence, however it is defined, and then circularly defines intelligence by whatever it is that the tests measure. One could have hundreds or thousands of such correlations at high levels, and yet have no real validation of whether what intelligence tests measure is in fact intelligence as opposed to some other cognitive construct, “general cognitive ability,” that is related to success in many academic as well as everyday-world endeavors.

Intelligence researchers have known for more than a century that all tests of general cognitive ability, whether or not it is “intelligence,” are highly intercorrelated [71]. One is assuming that they measure intelligence, but such an assumption is a large leap of faith—it is not tantamount to a proof, unless one makes the Boring-type circular argument that intelligence tests measure intelligence because they measure general cognitive ability, and intelligence is general cognitive ability because that is what the tests test. Factor-analytic studies provide internal validation for a hierarchical theory, but what is not as clear is, theory of what?

Many kinds of abilities tend to hang together, as in Carroll’s model and the CHC model, as described above, and even in the more radical work of Howard Gardner [72]. The fact that people who have certain high skills in a given domain tend to have high skills in other aspects of that domain suggests that the skills form a general factor of something, but not necessarily a general factor of intelligence as adaptation.

A second criterion against which intelligence tests are validated is school grades. But academic performance in school at any level is a weak criterion for validating a test of intelligence [73]. The tradition of using school grades as a criterion is probably in part an historical accident, stemming from Alfred Binet and Theodore Simon’s use of school performance as a built-in criterion for validating the usefulness of an intelligence test [74]. Further, school performance is, in a sense, “low-hanging fruit” in that it is easy to measure. In many cultures, school performance is not particularly valued or even may be devalued [75]. Of course, some cultures do not even have formal schooling, which in the course of human history, is a fairly recent invention. If one uses school grades as a criterion for validating an intelligence test, one is buying into a cultural value system—school grades are important and a valid indicator of intelligence—that may not be all that widely shared across time and space. Moreover, success in getting elevated school grades certainly depends on much more than intelligence and may depend more on traits that are counter to what may lead to success in later life, such as great skill in working in groups (forbidden on intelligence tests and in most schoolwork), than on working individually. In much of life, it is not enough or may even be counterproductive merely to do what one is told to do and do it well. Further, even where school grades are used, their importance with regard to life success is at least questionable. There is an obvious difference between doing well in school and succeeding, in any meaningful sense, for the remainder of one’s life. For some valedictorians, being first in their graduating class is their last major life success.

A third criterion against which intelligence tests have been validated is age. It makes sense that raw intelligence would increase with age. But so do height, weight, and number of adult teeth. Of course, at some point, these all stop increasing, but then, so apparently does intelligence. Thus, age, at least up to age 18 years or so, would seem to be a reasonable but weak criterion against which to validate an intelligence test.

A fourth criterion constitutes a variety of measures of professional success. Once again, these criteria are extremely culturally bound. Is annual income important, and if so, to whom? Is success in all jobs an important indicator of intelligence, or just some jobs, or really, any jobs? What value system leads some jobs (e.g., lawyer) to be viewed as more highly related to intelligence than other jobs (e.g., farmer), a situation that might be reversed in another culture? How is success to be measured? If an individual has life goals that do not lead to a prestigious job, high income, or high supervisory ratings but that leads to that individual’s helping many people and perhaps saving lives, how does one measure the success of that individual? Teachers, for example, are underpaid and underappreciated, but the future of our children depends on them. Occupational success is a matter of cultural convention. Even within a culture, some might view success differently; for example, is a lawyer who gets murderers off the hook for their crimes on technicalities “successful”, and if so, in what sense?

Obviously, there is no one perfect criterion and so it makes sense to use multiple criteria. One might argue that even though no one criterion is perfect, the fact that scores on intelligence tests correlate with a number of different high-stakes variables shows that they measure intelligence. But the criteria that are typically reported in test manuals and published research articles are all culturally bound and so it is not clear that they are the rights ones against which to validate intelligence tests, at least, to the extent to which one is concerned with biologically adaptive intelligence. Besides, what do such correlations actually show? Height correlates with many criteria of success but it does not therefore become “intelligence”.

Suppose, though, that one acknowledges that there is a paradox of supposedly intelligent people acting in ways that are species-destructive or even destructive to the future of their own gene pool. How could one explain such behavior?

### 3.3. Intelligence Is Ultimately Self-Destructive

One explanation might be that intelligent life, by its nature, is ultimately self-destructive—that once it reaches a certain point in the development of civilization, it turns on itself and destroys itself [76,77]. This explanation has been offered as a resolution to the Fermi paradox [78] of why, given the vastness of the universe, alien species have not landed on Earth. The reason given is that species are ultimately self-annihilating, for example, with industrialization causing climate change and related problems that ultimately kill them off before they reach the point of interstellar space travel. Hart [79] refers to this as the self-destruction hypothesis.

The problem with the self-destruction hypothesis is that it fails to explain how intelligence, which by definition is adaptive, ultimately can be fatally maladaptive for any advancing species. If a set of skills is actually maladaptive, then it seems that almost any label would apply better to the set of skills than the label of “intelligence,” which by definition is adaptive to the environment.

### 3.4. Constructs Other Than Intelligence Explain Why People Act Against Their Biological Interests

Another possibility is that constructs other than intelligence would be best called upon to explain why people act against their own biological or other interests. Examples might be avarice, as mentioned earlier, or narcissism, Machiavellianism, or psychopathy. It may be that the best construct for understanding people’s self-destructiveness may be wisdom [80,81,82], or rather, lack of wisdom—foolishness [83], or even opposition to wisdom—toxicity [84]. On this view, someone can be intelligent but unwise, foolish, or even toxic. Toxic leaders, for example, often encourage people to act against their best interests and often, as well, to act in ways that harm the natural environment upon which future generations will depend.

Ultimately, I have tried to resolve the paradox by including wisdom within my augmented theory of successful intelligence [85] so that people who work to harm the environment for themselves, their progeny, and humanity in general, would get “dinged,” with respect to evaluations of their level of intelligence, for their support of biological maladaptive causes. This theory goes beyond general intelligence in arguing that, in order to be fully adaptive, intelligence requires not only the analytical skills that constitute general intelligence, but also creative, practical, and wisdom-based skills. On this view, if a person’s creative, analytical, or practical skills are used for dark ends see [86,87], that would be reflected in a reduced evaluation of the person’s overall level of successful intelligence.

This solution is, I believe, only partially successful. The reason is that someone who acts against his or her own as well as others’ biological adaptive interests still can come out fairly highly on the intelligence scale. This is true despite that individual’s acting in ways that are blatantly maladaptive, unless wisdom is made a necessary condition for high levels of successful intelligence. If, for example, someone were highly creative in devising a weapon that could destroy all of human civilization, highly analytical in making sure it works, highly practical in ensuring that it can be delivered flawlessly, but extremely unwise in having undertaken the project in the first place, they still might be viewed as, on the whole, quite successfully intelligent, despite their potentially destroying all of humanity—or perhaps actually committing the act. Such a compromise might be a step in the right direction, but only a step.

### 3.5. Intelligence as Individual, Not Species Adaptation

Another explanation of how someone could have so-called “high intelligence” and yet act in biologically destructive, maladaptive ways is to posit that intelligence has nothing to do with species-based genetic adaptations. Rather, on this view, intelligence is relevant only to individual adaptation. In other words, intelligence is about how well individuals adapt to their personal environments, not about collective adaptations of any kind. Following this line of argument, one might claim that it is for this reason that the criteria used are all related to individual adaptation.

This explanation for species-maladaptive behavior is lacking, I believe, because, as discussed above, the explanation would apply only if intelligence is defined solely culturally, not biologically. If intelligence is merely about individual preservation, then intelligence is not a biologically-based attribute in a Darwinian sense. That said, acting in ways that increase global climate change, air pollution, microbial antibiotic resistance, or in other ways that actually directly harm the individual, not just his or her species or progeny, scarcely seems to be individually adaptive. Working for a polluting company may increase one’s income but potentially at the expense of one’s own and others’ health. If that is intelligence, it seems to be so in a very limited, culturally-defined sense. Of course, there are people who work for such companies because they have no other choices. They may lack the education or resources to find other jobs. But those individuals with more resources often have other options.

One could argue, of course, that this discussion of genetic and species preservation reflects values, and that intelligence in a biological sense must be value-free. But that is precisely the problem not with a biological conception of intelligence but rather with the current psychometric conception. It defines certain culturally approved behavior as “intelligent,” but what societies consider intelligent behavior varies widely across cultures. What is biologically common to all cultures is biologically—based adaptation.

Individual adaptation does not really have a biological definition, aside, perhaps, from preservation of one’s own life and passing one’s genes to the next generation. But it is hard to imagine a definition of intelligence that stops with preservation of one’s own life or set of genes. Indeed, it would be impossible to separate such a definition from a moral judgment, for example, if one had to choose between one’s own life and those of hundreds or thousands or millions of other people. Further, biological conceptions of intelligence certainly need to go beyond such moral judgments about how much one’s own life is worth versus that of some number of others.

In short, individual adaptation is a biologically vacuous concept. Evolutionary biologists scarcely pay attention to individuals, because their goal is to understand adaptation at collective levels, such as of species or phyla. The focus that psychologists—at least, North American and European ones—have placed on individual adaptation reflects a strong cultural bias. It reflects a bias toward individualism as opposed to collectivism [88,89], which, globally, probably reflects a minority position. In many societies, individuals are viewed as of little importance beyond serving collective interests. For example, some African and especially Asian societies tend to be more collectivist than are European and North American ones [90]. In a collectivist culture, criteria for the validity of a test of intelligence emphasizing individual accomplishments or gains might well seem oxymoronic, and individuals in collectivist cultures might even have difficulty taking intelligence tests that view collaboration as unethical or otherwise wrong [91,92]. The triumph of individualism in our conception of intelligence is shown by the fact that someone who is in a prestigious, high-paying managerial job in which he destroys the environment (for example, his job involves polluting the ocean) might well be adjudged by the usual culturally-based criteria to be generally intelligent rather than generally unintelligent. The issue is not which orientation is somehow “correct” or “better” but rather that cultural values perhaps should not play such a great role in a biological definition of intelligence.

## 4. Is the Western Conception of “Intelligence” Actually Intelligence?

If we now look at the available evidence, the assertion that so-called *g-*related phenomena are truly intelligence is based on a house of cards.

First, the fact that IQ tests and subtests intercorrelate with each other only tells us that they measure related cognitive (and other) functions. These intercorrelations do not tell us that those functions are intelligence.

Second, the fact that IQ tests yield a general factor and a hierarchical structure under *g* also does not tell us that the tests measure intelligence, at least in any biological sense. This fact tells us that the tests measure a series of related cognitive and other abilities. The reification of these abilities into “intelligence” is simply an assertion.

Third, the fact that IQ tests correlate with many and diverse kinds of behavior [93] tells us that general cognitive ability has many real-world correlates that people in Western societies think are important, such as school grades. People in other cultural settings value other things, as discussed above. Clearly, for Western societies, what some call general cognitive ability is important culturally [94]. Nothing I say in this article is intended to dispute that. But is it intelligence in any biologically adaptive, as opposed to a particularly culturally adaptive, sense? That is not clear.

Fourth, some might take the discovery of brain correlates of general cognitive ability with brain functioning (see Note [5]) as dispositive that general cognitive ability is indeed intelligence. In fact, such correlations have no dispositive value at all. Any cognitive or, for that matter, emotional function will have brain correlates. Who, really, would dispute that cognitive activity originates in the brain, or at least, in the central nervous system? Behavior that once was linked to intelligence but is no longer so linked, for example, sensory behavior, also emanates in the central nervous system and is mediated in some way by the brain. The fact that behavior is brain-based does not mean it is “intelligent.” Almost all behavior, except reflexes, intelligent or not, is brain mediated in some way, including behavior that is unrelated to intelligence and behavior that we might call “stupid.”

So, then, what is intelligence in a biological sense? I return to the argument made at the beginning of this article—that the thought and behavior that is intelligent in a biological sense is intelligent by virtue of its being adaptive in a biological sense. It is adaptive intelligence. Virtually none of the various demonstrations by psychometricians and cognitive psychologists demonstrate that what they are calling “intelligence” is adaptive in a biological sense. Furthermore, behavior that is broadly adaptive in a biological sense helps a species (or gene pool) survive and thrive through narrow adaptation to, shaping of, and selection of environments that the organism faces. Humans are not necessarily the best adapters in a narrow sense—bacteria and cockroaches have been around longer and almost certainly will be around longer—but humans are unique in their superior ability to shape and select environments.

Unfortunately, humans often have used their cognitive abilities to shape the environment in biologically maladaptive ways. One could argue that human contributions to global climate change, air pollution, and bacterial resistance to antibiotics tell us far more about human intelligence—or the lack thereof—than do human scores on tests of IQ. Why is the ability to solve a number-series problem a better indicant of “intelligence” than the ability to think of ways to mitigate worldwide disaster? Why are school grades more important criteria against which to measure intelligence than decisions that could make the difference between environmental preservation and destruction? Should not we be emphasizing, in our schools, the development of adaptive intelligence at least as much as the development of general intelligence, in that the former is what might help ensure that our progeny are able to benefit from whatever contributions we make to the world?

Some might say that issues like mitigating global climate change or air pollution are political, moral, or values issues. It is not clear, though, why preventing the destruction of the species would be a values issue but deciding to ask what number comes next in a series of numbers or what a rotated shape looks like is not. What value system leads one to decide that solving number-series problems or solving spatial problems unlike any one will encounter in one’s life, is more relevant to intelligence than the ultimate in biological adaptation—preservation of one’s life and health, the life and health of one’s progeny, and the life and health of *Homo sapiens?* If we view intelligence as adaptive, if we wish then to speak of adaptive intelligence, then we will learn little about it from the cultural artifacts that have been proposed as criteria for understanding intelligent thought and behavior. Rather, we should turn to biologically-based behavior that preserves the world for ourselves and for future generations and that recognizes the relation between biology and the ecological niches in which we (and other species) live [95,96,97].

The main points I have made in this article are not unique to me. Those who study the extremes of intelligence have long held similar ideas. Consider both ends of the intellectual spectrum.

At the lower end, the American Association on Intellectual and Developmental Disabilities (AAIDD), formerly the American Association on Mental Retardation (AAMR), has long defined intellectual disability in terms of both what I have called in this article “general intelligence” and in terms similar to what I have called “practical intelligence,” which is closely related to adaptive intelligence. In particular, the association defines “intellectual disability [a]s a disability characterized by significant limitations in both intellectual functioning and in adaptive behavior, which covers many everyday social and practical skills. This disability originates before the age of 18,” (https://aaidd.org/intellectual-disability/definition). In this definition, “intellectual functioning—also called intelligence—refers to general mental capacity, such as learning, reasoning, problem solving, and so on.” Furthermore, “adaptive behavior is the collection of conceptual, social, and practical skills that are learned and performed by people in their everyday lives.” They do not use terms such as “practical intelligence” or “adaptive intelligence,” but clearly are referring to skills that they view as different from those comprising general intelligence. On this view, similar to what I have argued for in the theory of successful intelligence, one cannot understand people’s intellectual functioning without going beyond the concept of *g.* This idea was proposed years ago, by one of the great leaders in the field of intellectual disability, Edward Zigler [98,99].

At the upper end, the National Association for Gifted Children (NAGC) has noted that “children are gifted when their ability is significantly above the norm for their age…. Giftedness may manifest in one or more domains such as; intellectual, creative, artistic, leadership, or in a specific academic field such as language arts, mathematics or science” (http://www.nagc.org/resources-publications/resources/what-giftedness). The NAGC, like the AAIDD, has recognized that a broad definition of intellectual functioning needs to go beyond general intelligence. Adaptation to the environment requires more than *g,* or IQ, or related constructs. It requires a broader conception of intelligence, referred to in this article as adaptive intelligence. If society appreciates the importance of broad and not just narrow adaptation, it may be in a better position to head off the destructive path on which humanity has set itself with regard to global climate change, pollution, weaponry, and related misadventures.

Of course, one could argue that, in a culture which believes that certain actions are good for humanity, perhaps burning coal, it is more adaptively intelligent to believe in the positive value of burning coal. Indeed, people have had many false beliefs over the years, such as that fat rather than sugar is the major cause of obesity [100]. In these cases, it may have been adaptively intelligent to accept the best scientific evidence available at the time. However, today, ample evidence exists for understanding most, although certainly not all of the causes of environmental destruction (and even of obesity), and the major question we face is why people ignore the facts that will save them and future generations [101]. In other words, why do they, whether of high or low IQ, fail to be adaptively intelligent in the face of facts that are readily available, should they seek those facts?

## 5. Implications for Action

There are at least three implications of the proposed theory for action.

First, psychologists in particular and society in general need to think more about what they mean by “intelligence.” If one is to take the notion of intelligence as adaptation seriously, then general intelligence may be general across a number of domains and yet not general when it comes to propagation of our species or gene pools. This lack of generality might matter less in a world not threatened by products of general intelligence—climate change, air and water pollution, weapons of mass destruction, etc.—but it does matter in the world in which we live.

There are, of course, many new theories of intelligence, such as Geary’s [102] theory of the role of mitochondria in intelligence, and the process-overlap theory of Kovacs and Conway and the Shipstead, Harrison, and Engle theory of the role of working memory and fluid intelligence as controlling maintenance and disengagement in intelligence (as cited earlier in this article). What perhaps is needed in these and other excellent theories is more discussion of the specific role of both biological and cultural adaptation to the environment as it relates to the biological and cognitive elements of these theories.

Second, educational and other institutions might wish to be more reflective in the skills they assess. Overwhelmingly, they emphasize cognitive and educational skills that are associated with individual success as traditionally defined in Western schooling but that might have less importance for sustaining the world as we have known it and as we might want to know it if we care about the preservation of our own and millions of other species. Tests that measure creative, practical, and wisdom-based skills [103,104]. The construct validity of these assessments needs further to be demonstrated before they are ready for high-stakes implementations.

Third, educational institutions might want to reconsider what and how they teach. There presently are teaching methods that teach beyond traditional kinds of pedagogy, for example, creative, analytical, practical, and wisdom-based learning and thinking [105,106]. Teaching for the liberal arts helps develop broad knowledge, but whether one learns to think creatively, analytically, practically, and wisely depends not just on what is taught, but also on how it is taught and what is actually learned from that teaching.

At present, the curriculum is highly skewed toward teaching for tests that measure proxies of general intelligence [107,108]. Perhaps schools should have a responsibility for teaching skills that develop not only general intelligence but also adaptive intelligence [109,110], the kind of intelligence that the world needs if it is to be preserved as a place in which humans can live and thrive.

## 6. Conclusions

San Bushmen, who have been in existence for over 100,000 years, and other hunter-gatherers, live in ways that involve adapting to and even shaping environments but that are sustainable and that are in harmony with nature [111]. In contrast, some human societies act, in many respects, like an invasive species, despoiling the environments they enter and destroying the quality of life that future generations otherwise might have enjoyed. About 17% of the Amazonian rainforest has been destroyed in the last 50 years (https://www.nationalgeographic.com/environment/habitats/rain-forests/) and the rate of deforestation is increasing, at the same time contributing substantially to emissions of carbon dioxide and global climate change [112]. The Bushmen, like so many other peoples, have recognized the importance of collective adaptation—that individual adaptation is not sufficient for a society successfully to adapt to the vagaries of nature. If our contemporary Western societies do not achieve the same recognition—if, whatever their general intelligence, they lack the adaptive intelligence to maintain a hospitable world for future generations, all the general intelligence in the world will not save the human species from extinction. Many young people already recognize these facts, as shown by the school strikes for climate on March 15, 2019, and September 20, 2019 [113]? Will the older generation recognize them before it is too late?
